# BRAF^V600E^-mutant metastatic NSCLC: disease overview and treatment landscape

**DOI:** 10.1038/s41698-024-00552-7

**Published:** 2024-04-16

**Authors:** David Planchard, Rachel E. Sanborn, Marcelo V. Negrao, Aria Vaishnavi, Egbert F. Smit

**Affiliations:** 1 Thoracic Cancer Group, Department of Medical Oncology, Gustave Roussy, Villejuif, France; 2 Earle A. Chiles Research Institute, Providence Cancer Institute, Portland, OR USA; 3 Department of Thoracic/Head and Neck Medical Oncology, Division of Cancer Medicine, University of Texas MD Anderson Cancer Center, Houston, TX USA; 4 Department of Cancer Biology, University of Texas MD Anderson Cancer Center, Houston, TX USA; 5 Department of Pulmonary Disease, Leiden University Medical Centre, Leiden, Netherlands

**Keywords:** Non-small-cell lung cancer, Targeted therapies

## Abstract

In this review, we cover the current understanding of *BRAF* mutations and associated clinical characteristics in patients with metastatic NSCLC, approved and emerging treatment options, *BRAF* sequencing approaches, and unmet needs. The BRAF^V600E^ mutation confers constitutive activity of the MAPK pathway, leading to enhanced growth, proliferation, and survival of tumor cells. Testing for *BRAF* mutations enables patients to be treated with therapies that directly target BRAF^V600E^ and the MAPK pathway, but *BRAF* testing lags behind other oncogene testing in metastatic NSCLC. Additional therapies targeting BRAF^V600E^ mutations provide options for patients with metastatic NSCLC. Emerging therapies and combinations under investigation could potentially overcome issues of resistance and target non-V600E mutations. Therefore, because targeted therapies with enhanced efficacy are on the horizon, being able to identify *BRAF* mutations in metastatic NSCLC may become even more important.

## Introduction

Mutations in the v-Raf murine sarcoma viral oncogene homolog B (*BRAF*) gene have been found in ~4–8% of all cancers, with the greatest number found in colorectal cancer (CRC), melanoma, and non-small cell lung cancer (NSCLC)^1–3^. The most common *BRAF* mutation is a point mutation (T1799A) resulting in an amino acid substitution at codon 600 (V600E), which confers constitutive BRAF kinase activity^2,4,5^. BRAF^V600E^ accounts for ~1–2% of NSCLCs, making it an actionable therapeutic target given the success of other therapies that target actionable mutations with similar frequencies in NSCLC (e.g., *ALK*, *EGFR*)^6–9^. Targeted therapeutic approaches with BRAF inhibitor monotherapy (vemurafenib and dabrafenib) demonstrated efficacy in phase 2 trials with generally acceptable toxicity in patients with BRAF^V600^-mutant advanced NSCLC^10,11^.

While BRAF^V600E^ inhibitor monotherapy is initially effective, acquired resistance and paradoxical activation are associated with reactivation of the mitogen-activated protein kinase (MAPK) pathway and subsequent disease progression^12–14^. To delay onset of resistance, BRAF inhibitors were combined with a downstream inhibitor of the MAPK pathway, MAPK kinase (MEK)^14^. Trametinib, a MEK inhibitor, in combination with dabrafenib showed durable anti-tumor activity and acceptable safety in patients with BRAF^V600E^-mutant metastatic NSCLC in phase 2 trials^15–17^. The combination of BRAF inhibitor encorafenib plus MEK inhibitor binimetinib is being investigated in ongoing phase 2 trials in patients with BRAF^V600E^-mutant metastatic NSCLC^5,18,19^. Initial results of the PHAROS trial revealed the combination had substantial and durable anti-tumor activity and a manageable safety profile^18^. Based on the results from this study, in October 2023, the US Food and Drug Administration (FDA) approved encorafenib plus binimetinib for patients with BRAF^V600E^-mutant metastatic NSCLC^20^. Current guidelines recommend dabrafenib plus trametinib or encorafenib plus binimetinib as preferred first-line treatment options or as subsequent treatment for BRAF^V600E^-mutant metastatic NSCLC^21^. BRAF monotherapy could be considered in certain circumstances, such as lack of tolerability.

While there has been notable progress in effective treatments for BRAF-mutant NSCLC^17,18^, several uncertainties remain. Current guidelines for BRAF^V600^-mutant NSCLC recommend BRAF-targeted therapy in the first-line setting, but the optimal course for patients who do not tolerate or progress while on first-line BRAF and MEK inhibitor combinations remains ambiguous^21,22^. Second-line recommendations include immunotherapy, chemotherapy, or a combination; however, immunotherapy data are limited and conflicting for patients with BRAF^V600^-mutant NSCLC^22–24^. Current targeted therapeutic approaches have limited efficacy in patients with non-V600 *BRAF* mutations, and most clinical trials have focused primarily on the V600E mutation since its discovery^11,17,18,25^. There remains a need to better understand the incidence, impact, and management of brain metastases; mechanisms of resistance; optimal sequencing; and other patient- (e.g., smoking history) or disease-related factors (e.g., PD-L1 expression) that influence treatment outcomes of BRAF-mutant metastatic NSCLC. This manuscript provides a review of BRAF-mutant metastatic NSCLC and the therapeutic landscape with particular emphasis on targeted therapies for the V600E mutation.

## BRAF-mutant metastatic NSCLC disease overview

### Clinical characteristics

While *BRAF* mutations are predominantly found in adenocarcinomas (>85%), there is no clear association of *BRAF* mutation status with other patient characteristics, such as age, ethnicity, and sex^6,8,9,26^. Epidemiological patterns are difficult to identify since *BRAF* mutations occur in a small number of patients with advanced NSCLC^5^. One study reported that BRAF^V600E^ mutations were significantly more common in females (*P* < 0.001), but that finding was not consistent with other studies^6,10,26^. While data are limited, studies have also shown that the incidence of *BRAF* mutations is lower in Asian patients than white patients^6,26,27^. Smoking history can be associated with *BRAF* mutations, as well as *KRAS, MET*, and other mutations^9,28,29^. However, a few studies have also suggested that BRAF^V600E^ is less associated with smoking history than other *BRAF* mutations^26,30,31^. Therefore, all patients with advanced NSCLC regardless of smoking history should undergo broad-based mutation testing, including *BRAF*^5,21,22^. Programmed death ligand 1 (PD-L1) expression and tumor mutation burden (TMB) are potentially important indicators of immunotherapy response and are frequently elevated in BRAF-mutant NSCLC^24^. The prognostic implication of *BRAF* mutation status is inconclusive due to small patient numbers and conflicting studies^8,26,28^. However, a few studies reported worse outcomes with chemotherapy in patients with V600E mutations versus wild type (WT) *BRAF* and conflicting results for V600E versus non-V600E mutations^6,31–33^. In addition to the small patient number, discrepancies in patient characteristics and prognosis could be attributed to the heterogeneity of *BRAF* mutations in NSCLC^33,34^.

### Classification of *BRAF* mutations

**Fig. 1:**
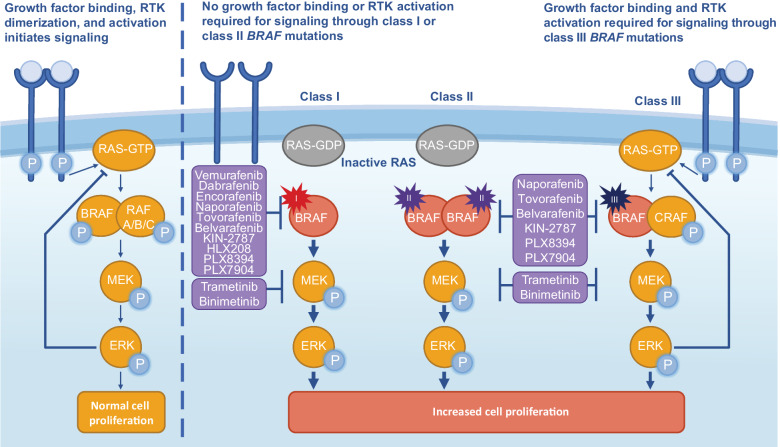
*BRAF* mutation classes and mechanism of actions for BRAF/MEK inhibitors. Class I and II mutations are RAS-independent, constitutively active monomers (class I) or dimers (class II). Class III mutations are RAS-dependent dimers with compromised kinase activity. Current BRAF inhibitors are effective for class I-mutant monomers. Next-generation RAF inhibitors can inhibit dimers and may inhibit class II and III mutations. P, phosphorylation.

*BRAF* mutations are heterogenous with distinct mutation classes that each yield a functionally diverse BRAF protein, resulting in different clinical features and treatment ramifications^33,35^. BRAF-mutant proteins interact with and activate the MAPK pathway in various ways and to different degrees (Fig. 1)^25,35,36^. In the MAPK pathway, the signaling cascade begins with growth factor binding to receptor tyrosine kinase (RTK) which facilitates RAS binding GTP^14,25,36^. Activated RAS promotes a cascade of activation starting with RAF family kinases (ARAF, BRAF, CRAF), which form activated RAF homo- or heterodimers. RAF kinases activate MEK, which subsequently activates extracellular signal-regulated kinase (ERK). ERK has multiple downstream targets, including transcription factors that promote cell growth, proliferation, and survival. Negative feedback loops from ERK also regulate the pathway^25^.

There are over 200 identified *BRAF* mutations categorized into three classes based on dimerization status, RAS-dependence, and kinase activity level^5,37^. Class I *BRAF* mutations occur on codon 600 (BRAF^V600^), substituting the valine to a glutamic acid, lysine, aspartic acid, arginine, or methionine (V600E, V600K, V600D, V600R, and V600M mutations, respectively) and can biochemically transform BRAF into a RAS-independent constitutively active monomer^25,36^. BRAF^V600E^ is the most prevalent class I mutation and accounts for ~30–50% of all *BRAF* mutations in NSCLC^6,7,33^. Class I mutant proteins have a high level of kinase activity, and BRAF^V600E^ is ~500-fold times more active than WT BRAF, leading to increased cellular proliferation^4,36^. Class I mutations all occur at codon 600 aberrantly activating monomers, but class II and III mutations occur at various other non-600 codons and form dimers^5,25,36^. Class II *BRAF* mutations span from G464 to K601, which includes the activation segment and P-loop domains of BRAF. These mutations, along with fusions and in-frame deletion mutations, are shown or predicted to be able to biochemically transform BRAF into a RAS-independent constitutively active dimer with ranges of intermediate to high kinase activity. Class III mutations, which tend to have impaired kinase activity, occur in the P-loop, catalytic loop, or DFG motif to form RAS-dependent heterodimers with CRAF^WT^. These mutations require upstream activation of RAS to amplify downstream signaling, similar to its normal physiological role and function.

Although constitutive activity of class I and II mutations can suppress RAS through ERK-negative feedback loop, class III mutations only mildly activate ERK, resulting in minimal negative feedback of RAS^25,38^. To overcome that feedback inhibition, class III *BRAF* mutations are often observed with concurrent *RAS* activating mutations^25,33,38^. While these coexisting mutations often occur in melanoma cancers, CRC and NSCLC cancers have fewer cases^25,38^: one study in NSCLC tumors reported coexisting *RAS* mutations in 22% of class III mutations (*n* = 54), including 42% of kinase-dead mutations (*n* = 19)^33^. This may be explained by basal RTK activation and subsequent RAS activity that is sufficient to support these class III mutants^25^.

Currently approved BRAF inhibitors effectively inhibit only class I mutant proteins and show substantially less efficacy against BRAF*-*mutant dimers (Fig. 1)^25^. BRAF monomer inhibitors binding to dimers can cause paradoxical transactivation of the unbound RAF^WT^ protomer, enabling MEK/ERK signaling and subsequent disease progression^12,13,25^. Upstream inhibition may be an effective therapeutic strategy for treatment of class III mutations^25,33,38^. A better understanding of *BRAF* mutations, especially class II and III mutations, may enable the rational design of new targeted therapies and the development of next-generation drug combination strategies to treat BRAF-mutant cancers, including NSCLC^25,33^.

### *BRAF* testing

Guidelines recommend that all patients with advanced non-squamous NSCLC undergo broad-based molecular testing to identify molecular drivers—including but not limited to BRAF^V600^ mutations^21,22^. Recommended and approved molecular testing assays include polymerase chain reaction (PCR) and next-generation sequencing (NGS)^39–41^. PCR offers rapid turn-around, reproducibility, specificity, sensitivity, and lower costs, but it is a single-gene assay typically limited to detection of V600E mutation^39^. Panel-based NGS has gained popularity for the ability to simultaneously test multiple genes, including *BRAF*, for both V600E and non-V600E mutations, which is more cost-effective than sequential single-gene assays and uses relatively little tumor tissue^39,40^. Availability of sufficient tumor tissue is a major constraint when testing for the numerous actionable mutations in NSCLC, so approaches that conserve tissue while providing a full molecular profile are being evaluated^42^. Liquid biopsy, a noninvasive and more rapid alternative to tissue biopsies that collects blood-based biomarkers—including circulating tumor DNA (ctDNA), can be used to detect genomic alterations^5,43^. Immunohistochemistry (IHC) is a highly sensitive and specific diagnostic test that uses the monoclonal antibody VE1 to detect BRAF^V600E^-mutant proteins^39,44^. While there are no current standard recommendations for IHC in BRAF^V600E^ mutation detection, guideline recommendations for other oncogenes suggest that IHC be confirmed with a molecular test^22,39^. *BRAF* testing rates lag behind other driver oncogene testing rates, likely due to limited tissue availability and the fact that other actionable biomarkers (e.g., *EGFR*, *ALK*, PD-L1) are prioritized for testing when a sequential selective approach is used^45^. Given the demonstrated efficacy of BRAF^V600E^ inhibitors in NSCLC, there is a need to improve the rate of *BRAF* testing so the results can be applied to clinical decision-making.

## Treatment landscape

### BRAF and MEK inhibitors in solid tumors

**Fig. 2:**
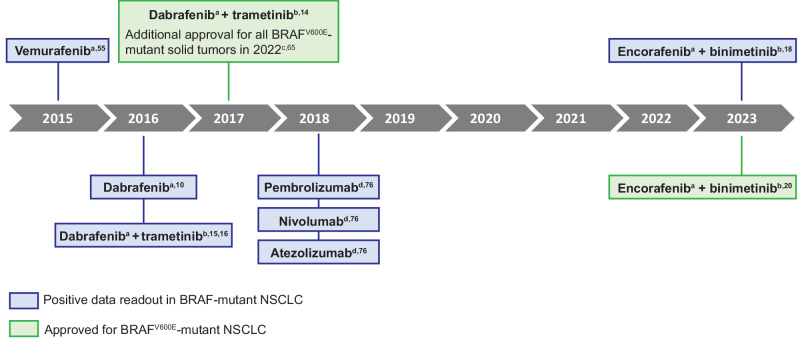
Timeline of key advancements in the treatment of BRAF^V600E^-mutant NSCLC. Positive data readouts for BRAF-mutant NSCLC (blue) and approved treatments for BRAF^V600E^-mutant NSCLC (green) are shown. ^a^BRAF inhibitors. ^b^MEK inhibitors. ^c^In 2022, dabrafenib plus trametinib was approved for patients with unresectable metastatic BRAF^V600E^-mutant solid tumors who progressed on previous treatments and have no acceptable alternative option. ^d^Immunotherapy that targets PD-1. ^e^Immunotherapy that targets PD-L1.

The identification of *BRAF* mutations, especially V600E, and their role in cancer led to the development of highly-selective BRAF inhibitors such as vemurafenib, dabrafenib, and encorafenib (Figs. 1 and 2) ^46–48^. These small-molecule inhibitors preferentially bind to the active conformation of BRAF kinase; through competitive occupation of the ATP binding pocket, the drugs stabilize the active conformation, resulting in potent inhibition of BRAF^V600^ ^47–49^. Both vemurafenib and dabrafenib have confirmed activity against V600E, V600K, V600R, and V600D *BRAF* mutations ^50–52^. Encorafenib has confirmed activity against BRAF^V600E^ and BRAF^V600K^ mutants and BRAF^WT^ ^48,49^.

BRAF inhibitor monotherapy has had varying degrees of efficacy in solid tumors^53–55^. In patients with BRAF^V600E^-mutant metastatic melanoma, single-agent vemurafenib was associated with a relative reduction of 63% in the risk of death and 74% in the risk of tumor progression compared with dacarbazine; median progression-free survival (PFS) was 5.3 months with vemurafenib (*n* = 275) and 1.6 months with dacarbazine (*n* = 274)^53^. With single-agent dabrafenib, patients with BRAF^V600E^-mutant metastatic melanoma (*n* = 76) had an objective response rate (ORR) of 59%, median PFS of 6.3 months, and median overall survival (OS) of 13.1 months^56^. In the dose expansion phase of a single-agent encorafenib study in patients with BRAF^V600^-mutant melanoma, ORR was 60.0%, median PFS was 12.4 months (95% CI, 7.4–not estimable [NE]), and median OS was NE for BRAF inhibitor-naive patients (*n* = 15); corresponding data for BRAF inhibitor-pretreated patients (*n* = 18) were 22.2%, 1.9 months (95% CI, 0.9–3.7), and 9.07 months (95% CI, 3.68–10.84)^57^.

A basket study evaluated vemurafenib monotherapy in patients with nonmelanoma BRAF^V600^-mutant cancers^55^. NSCLC and CRC cohorts had ORRs of 42% (95% CI, 20–67%) and 0%, respectively (Table 1). The poor clinical activity of BRAF inhibitor monotherapy in patients with CRC was consistent in additional vemurafenib (5% partial response [PR]; *n* = 21), dabrafenib (11% PR; *n* = 9), and encorafenib studies (0% PR; *n* = 18)^54,58,59^. In patients with BRAF^V600^-mutant CRC, addition of cetuximab, an anti-EGFR-antibody, improved ORR with vemurafenib (4%; *n* = 27) and encorafenib (19.5%; *n* = 220)^55,60^.

**Table 1:** Selected trials of BRAF and MEK inhibitors

Treatment	Mutation status	Patients, *n*	Median follow-up, mo	ORR (CR/PR), %	Median DOR, mo	Median time to response, mo	Median PFS, mo	Median OS, mo	Safety	Interruption, reduction, and permanent discontinuation, %^a^	Ref
*BRAF Inhibitor Monotherapy*											
Vemurafenib	V600E (90%), V600G (5%), unknown V600 (5%)	20^b^ (treatment-naive and previously treated)	NA	42 (0/42)	NA	NA	7.3	NE	Grade 3/4 AEs: 80% Common any-grade AEs: decreased appetite (35%), nausea (35%), dyspnea (30%), hyperkeratosis (30%), vomiting (30%)	NA	55
Vemurafenib	V600E (98%), unspecified V600 (2%)	8 (treatment-naive)	10.7	37.5 (0/38)	NE	NE	12.9	NE	Grade 3/4 AEs: 77% Common any-grade AEs: nausea (40%), hyperkeratosis (34%), decreased appetite (32%), arthralgia (31%)	40, 61, 10	66
		54 (previously treated)	10.7	37.0 (0/37)	6.1	7.3	6.1	15.4			
Vemurafenib	V600E (96%), V600K (2%), V600D (1%), V600M (1%)	101 (previously treated)	23.9	44.8 (NA/NA)^c^	6.4	NA	5.2	10	SAEs: 36% Common any-grade TRAEs: asthenia (56%), decreased appetite (46%)	NA, NA, 24	11
Dabrafenib	V600E	6 (treatment-naive)	NA	67 (0/67)	NA	NA	NA	NA	Grade 3/4 AEs: 44% Common any-grade AEs: pyrexia (36%), asthenia (30%), hyperkeratosis (30%), decreased appetite (29%), nausea (27%), cutaneous SCC (12%)	43, 18, 6	10
		78 (previously treated)	10.7	33 (NA/NA)	9.6	NA	5.5	12.7			
*BRAF and MEK Inhibitor Combination Therapies*											
Dabrafenib plus trametinib	V600E	36^d^ (treatment-naive)	15.9	64 (6/58)	10.4	NA	10.9	24.6	Grade 3/4 AEs: 69% Common any-grade AEs: pyrexia (64%), nausea (56%), diarrhea (36%), fatigue (36%), peripheral edema (36%), vomiting (33%), dry skin (33%), decreased appetite (33%)	75, 39, 22	16
Dabrafenib plus trametinib	V600E	57^e^ (previously treated)	11.6	63.2 (3.5/59.6)	9.0	1.4^f^	9.7	NE	Grade 3/4 AEs: 49% Common any-grade AEs: pyrexia (46%), nausea (40%), vomiting (35%), diarrhea (33%), asthenia (32%), decreased appetite (30%), cutaneous SCC (4%)	61, 35, 12	15
Encorafenib plus binimetinib (PHAROS)	V600E^g^	59 (treatment-naive)	18.2^h^	75 (15/59)	NE	1.9	NE	NE	Grade 3/4 TRAEs: 41% Common any-grade TRAEs: nausea (50%), diarrhea (43%), fatigue (32%), vomiting (29%) All-causality pyrexia (22%)	44, 24, 15	18
		39 (previously treated)	12.8^h^	46 (10/36)	16.7	1.7	9.3	NE			

*AE* adverse event, *CR* complete response, *DOR* duration of response, *NA* not available, *NE* not estimable or not reached, *ORR* objective response rate, *OS* overall survival, *PFS* progression-free survival, *PR* partial response, *SAE* serious AE, *SCC* squamous cell carcinoma, *TRAE* treatment-related AE.

^a^Dose interruption, dose reduction, and permanent treatment discontinuation due to AEs.

^b^Efficacy and safety were analyzed for 19 patients. However, one patient dropped out prior to assessment of response and was included in the denominator for efficacy but as having no response.

^c^ORR was analyzed for 96 patients.

^d^Updated 5-year analysis reported ORR of 63.9%, median PFS of 10.8 months, and median OS of 17.3 months^17^.

^e^Updated 5-year analysis reported ORR of 68.4%, median PFS of 10.2 months, and median OS of 18.2 months^17^.

^f^Median time to response was reported as 6 weeks in the paper. Weeks to months was calculated with 1:0.23 conversion.

^g^One previously treated patient had both V600D and V600E mutations in their tumor.

^h^Median duration of follow-up for this study was for PFS.

While BRAF inhibitor monotherapy is initially effective, acquired resistance enables reactivation of the MAPK pathway, limiting the clinical utility of monotherapy^14,61^. In addition, BRAF monomer inhibitors can cause paradoxical activation of the MAPK pathway in BRAF^WT^ cells, which has been associated with hyperproliferative cutaneous events, including squamous cell carcinoma (SCC) and keratoacanthoma^12–14,57^. BRAF monomer inhibitors were combined with downstream MEK inhibitors to overcome resistance and paradoxical activation of the MAPK pathway, which increased efficacy and tolerability, resulting in several combination therapies being approved for unresectable metastatic BRAF^V600^-mutant melanoma (e.g., dabrafenib plus trametinib, vemurafenib plus cobimetinib, encorafenib plus binimetinib)^14,62–64^. The FDA granted accelerated approval of dabrafenib plus trametinib treatment for previously treated unresectable or metastatic solid tumors with the BRAF^V600E^ protein in patients with no alternative treatment options^65^. This approval was supported by the ROAR study, which enrolled 206 patients into eight cohorts, each a different BRAF^V600E^-mutant rare cancer. ORR ranged from 0% for gastrointestinal stromal tumor (*n* = 1) to 89% for hairy cell leukemia (*n* = 55); ORR was ≥33% for the remaining cohorts.

The safety profile of BRAF inhibitor monotherapy was similar across solid tumors; common adverse events (AEs) included arthralgia, fatigue, rash, cutaneous events (e.g., SCC, keratoacanthoma), and gastrointestinal issues (e.g., diarrhea, nausea)^10,48,53,54,57,62,64,66^. Drug-specific AEs include photosensitivity with vemurafenib, pyrexia with dabrafenib, and reduced incidences of SCC and keratoacanthoma with encorafenib. Adding a MEK inhibitor resulted in a few key differences in the safety profiles^62,64^: pyrexia was more frequent with dabrafenib plus trametinib versus dabrafenib monotherapy^15,62^ and hyperproliferative cutaneous events were less common with dabrafenib plus trametinib and encorafenib plus binimetinib^15,62,64^.

### BRAF and MEK inhibitors in metastatic NSCLC

Clinical trials with BRAF inhibitor monotherapy in patients with BRAF^V600^-mutant NSCLC are summarized in Table 1^10,11,55,66^. In several trials, vemurafenib monotherapy was an effective treatment for treatment-naive (ORR: 37.5%; median PFS: 12.9 months) and previously treated patients (ORR: 37.0–44.8%; median PFS: 5.2–6.1 months) with BRAF^V600^-mutant NSCLC^11,66^. In one study, serious AEs occurred in 63% of patients, most commonly cutaneous SCC (15%) and keratoacanthoma (15%)^66^. Dabrafenib monotherapy was effective for previously treated patients (ORR: 33%; median PFS: 5.5 months) with BRAF^V600E^-mutant metastatic NSCLC; however, data were limited for treatment-naive patients because of a decision to prioritize the combination with trametinib with the expectation of improved response rates^10^. Pyrexia was the most common any-grade AE (36%), including grade 3 occurrences in 2% of patients. Pyrexia led to dose interruption or reduction in 11% and 4% of patients, respectively. Serious AEs occurred in 42% of patients, including pyrexia in 6% of patients.

**Fig. 3:**
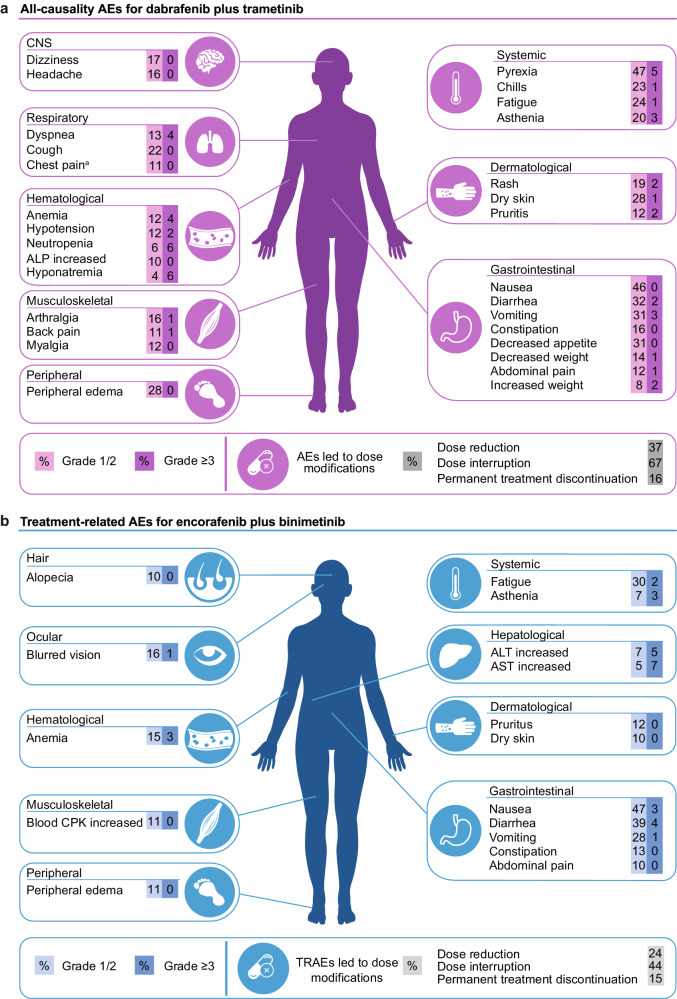
Adverse events experienced by ≥10% of patients with BRAF/MEK combination therapies. **a** Adverse events (AEs) shown for dabrafenib plus trametinib occurred in at least 10% of patients in combined data from interim analysis of treatment-naive and previously treated patients^15,16^. AEs led to dose reduction, dose interruption, and permanent treatment discontinuation in 37%, 67%, and 16% of patients. ^a^Chest pain includes musculoskeletal chest pain. **b** Treatment-related AEs (TRAEs) shown for encorafenib plus binimetinib occurred in at least 10% of patients^18^. TRAEs led to dose reduction, dose interruption, and permanent treatment discontinuation in 24%, 44%, and 15% of patients. Comparisons of safety profiles should be done cautiously since data are from different trials and reported as all-causality AEs for one treatment combination and as TRAEs for the other combination. ALP alkaline phosphatase, ALT alanine aminotransferase, AST aspartate aminotransferase, CNS central nervous system, CPK creatine phosphokinase.

The combination of BRAF and MEK inhibitors demonstrated synergistic anti-tumor activity with a manageable safety profile (Table 1)^14–16,18^. The multicenter, non-randomized, open-label, phase 2 trial evaluated dabrafenib plus trametinib in patients with BRAF^V600E^-mutant metastatic NSCLC^15–17^. At the initial data analysis with a median follow-up of 15.9 months, for treatment-naive patients (*n* = 36), ORR by investigator was 64% (95% CI, 46–79%), median duration of response (DOR) was 10.4 months (95% CI, 8.3–17.9), median PFS was 10.9 months (95% CI, 7.0–16.6), and median OS was 24.6 months (95% CI, 12.3–NR)^16^. The most common AEs included pyrexia (64%), nausea (56%), and diarrhea (36%); grade 3–4 AEs occurred in 69% of patients, including pyrexia (11%) (Fig. 3a). For previously treated patients (*n* = 57), with a median follow-up of 11.6 months, ORR by investigator was 63.2% (95% CI, 49.3–75.6%), median DOR was 9.0 months (95% CI, 6.9–18.3), median PFS was 9.7 months (95% CI, 6.9–19.6), and median OS was immature^15^. The most common AEs included pyrexia (46%), nausea (40%), and vomiting (35%); grade 3–4 AEs occurred in 49% of patients, including pyrexia (2%) (Fig. 3a). At the 5-year follow-up analysis, median PFS and OS were 10.8 months (95% CI, 7.0–14.5) and 17.3 months (95% CI, 12.3–40.2) for treatment-naive patients and 10.2 months (95% CI, 6.9–16.7) and 18.2 months (95% CI, 14.3–28.6) for previously treated patients, respectively^17^. The most common AE remained pyrexia (56%), and grade 3–4 AEs occurred in 66% of patients with most manageable with dose modifications. Pyrexia led to dose reduction in 11 patients (12%) and permanent treatment discontinuation in two patients (2%). The addition of the MEK inhibitor was associated with lower incidence of cutaneous SCC compared with BRAF inhibitor monotherapy (4% versus 12%)^10,15^. Based on these data, the FDA and European Medicines Agency approved dabrafenib plus trametinib combination for treatment of patients with BRAF^V600E^-mutant metastatic NSCLC^67,68^. A later retrospective analysis supported the use of dabrafenib plus trametinib, where the risk of death for treatment-naive patients with BRAF-mutant advanced NSCLC was significantly lower with dabrafenib plus trametinib versus platinum-based chemotherapy (HR = 0.51; 95% CI, 0.29–0.92; *P* = 0.03), and median OS was 17.3 months (95% CI, 14.6–NR) versus 9.7 months (95% CI, 6.4–19.6), respectively^68^.

The combination of encorafenib plus binimetinib is being investigated in ongoing phase 2 trials in patients with BRAF^V600E^-mutant NSCLC^5,18,19^. PHAROS, a single-arm, open-label, multicenter trial (NCT03915951), enrolled 98 patients with BRAF^V600E^-mutant metastatic NSCLC (*n* = 59 treatment-naive, *n* = 39 previously treated)^18^. In treatment-naive patients, with a median follow-up for PFS by independent radiology review (IRR) of 18.2 months (95% CI, 16.4–22.3), ORR assessed by IRR was 75% (95% CI, 62–85%), median DOR by IRR was NE (95% CI, 23.1–NE), and median PFS by IRR was NE (95% CI, 15.7–NE). In previously treated patients, with a median follow-up for PFS by IRR of 12.8 months (95% CI, 9.0–19.8), ORR by IRR was 46% (95% CI, 30–63%), median DOR by IRR was 16.7 months (95% CI, 7.4–NE), and median PFS by IRR was 9.3 months (95% CI, 6.2–NE). OS was NE in both groups. The most frequently reported treatment-related AEs (TRAEs) were nausea (50%), diarrhea (43%), fatigue (32%), and vomiting (29%); serious TRAEs occurred in 14% of patients with the most common being colitis (3%) (Fig. 3b). All-causality pyrexia occurred in 22% of patients, and treatment-related pyrexia led to one dose interruption and no dose reductions or permanent discontinuations. The ENCO-BRAF trial (NCT04526782) includes treatment-naive and previously treated cohorts, with an estimated enrollment of 119 patients to conclude in 2026^19^. Encorafenib plus binimetinib combination treatment was recently approved by the FDA for patients with BRAF^V600E^-mutant metastatic NSCLC based on the PHAROS trial^20^.

### Primary and acquired drug resistance to BRAF-targeted therapy

Baseline concurrent mutations prior to receipt of targeted therapy have been identified in 22–30% of patients with BRAF^V600E^-mutant NSCLC and may be a cause of primary resistance^17,61,69^. Common concurrent mutations included alterations in the *TP53* and *SETD2* genes and the PI3K (e.g., *PIK3CA* E545K, *PTEN* R14K) and MAPK (e.g., *KRAS* G12C) pathways^17,18,61,69^. The incidence of concurrent *TP53* or *RAS* gene family mutations was higher with *BRAF* class II or III mutations than class I mutations^31,33,70^. In several studies, the presence of a concurrent mutation in *TP53, PIK3CA, KRAS,* or *PTEN* was associated with poorer outcomes^17,61,69^. In a study evaluating dabrafenib plus trametinib for BRAF^V600E^-mutant metastatic NSCLC, patients with a concurrent mutation in the PI3K pathway (*n* = 4) had a median OS of 5.4 months compared with 22.7 months in patients without an identified concurrent mutation (*n* = 34)^17^. In the PHAROS trial, which evaluated encorafenib plus binimetinib for BRAF^V600E^-mutant metastatic NSCLC, concurrent mutations were identified, with the most common being *SETD2* and *TP53* (43%, each); however, there was no association between concurrent mutations and outcome^18^. As most of this data comes from studies with small numbers of patients, further research is necessary to understand the impact of concurrent mutations in patients with BRAF^V600E^-mutant NSCLC^17,18,69^.

The mechanisms of acquired resistance to BRAF inhibitors, alone or combined with MEK inhibitors, are poorly understood, and there is no standardized sequential treatment strategy^21,61^. While data are limited, acquired resistance appears to occur through bypassing or reactivating the MAPK pathway^14,61^. Bypassing the MAPK pathway and activating a parallel pathway (e.g., PI3K/AKT) can lead to disease progression. A *PTEN* frameshift mutation that could potentially activate the PI3K pathway was identified in a patient with BRAF^V600E^-mutant NSCLC that progressed on dabrafenib^71^. In preclinical BRAF^V600E^-mutant lung cancer models, the presence of a cooperating mutation silencing RBMS3, a regulator of the WNT pathway, promoted resistance to dabrafenib plus trametinib^72^.

Reactivation of the MAPK pathway can occur in BRAF-dependent or -independent manners. Resistance to targeted therapies often occurs due to secondary mutations or epigenetic changes in the target gene, and an aberrant splice variant of BRAF was identified in BRAF^V600E^ NSCLC cells that acquired resistance^73,74^. However, secondary *BRAF* mutations may be rare, as none were discovered in several resistance studies with BRAF-mutant NSCLC and melanoma^61,73,75^. BRAF-independent reactivation of the MAPK pathway includes alterations either upstream or downstream of BRAF^14^. Mutations in *RAS* (*NRAS, KRAS*) were discovered in a few studies^61,71^. Strong evidence came from a study that compared ctDNA sequencing at diagnosis and disease progression for 35 patients with BRAF-mutant NSCLC who progressed on either BRAF inhibitor monotherapy or dabrafenib plus trametinib^61^. *RAS* mutations (*KRAS* G12V; *KRAS* Q61R; *NRAS* Q61R) were present at disease progression and not diagnosis, which suggests mutation occurred during treatment. Resistance studies of patients with BRAF^V600E^-mutant melanoma suggested that upregulation of RAS or overexpression of ARAF and CRAF could alleviate BRAF-dependence in tumor cells^73,75^. Downstream mutations in MEK1 were also identified in patients with BRAF^V600E^-mutant NSCLC that progressed on dabrafenib plus trametinib^71^. Further understanding of acquired resistance mechanisms is critical to inform optimal sequencing and providing insight for evaluation of combination approaches or next-generation target therapies.

### Immunotherapy

Compared with studies with BRAF and MEK inhibitors, data concerning efficacy and safety of immunotherapy in patients with BRAF^V600E^-mutant NSCLC are limited; studies have not prospectively enrolled patients with BRAF^V600^ mutations, and immunotherapy is not specifically approved for patients with this mutation^76–79^. Evidence for efficacy of immunotherapy is derived from conflicting, small, retrospective studies (Table 2)^23,24,77^. In a multi-cohort retrospective study of immunotherapy in patients with oncogene-driven advanced lung cancer, patients (*n* = 10) with BRAF-mutant NSCLC in one cohort had a significantly longer median PFS (7.4 months) than patients with other common oncogene drivers (versus *KRAS* 2.8 months; HR 0.36, 95% CI, 0.14–0.88; *P* = 0.026)^24^. In the other cohort, PFS was longer in patients with V600E mutations (n = 30; 9.8 months) versus non-V600E mutations (*n* = 35; 5.4 months). However, in another retrospective study, similar PFS (2.1–3.4 months) was reported across oncogenes with immunotherapy^77^. In that study, two trends emerged in the BRAF cohort (*n* = 43). PFS was longer in patients who previously or currently smoked (4.1 months) versus had never smoked (1.9 months) and with non-V600E mutations (4.1 months) versus V600E mutations (1.8 months). According to guidelines of the European Society for Medical Oncology, patients with BRAF^V600^-mutant metastatic NSCLC that progresses on BRAF plus MEK inhibitor should receive immunotherapy with optional chemotherapy (in patients with smoking history) or chemotherapy with optional immunotherapy (in patients without smoking history)^22^.

**Table 2:** Efficacy data for chemotherapy and immunotherapy trials that included patients with BRAF-mutant NSCLC

Treatment	Patients, *n* (*BRAF* mutation)	Treatment status	Median follow-up, mo	ORR, %	Median PFS, mo	Median OS, mo	Ref
Platinum-based doublet chemotherapy^a^	7 (V600E)	Treatment-naive	13.7	29	4.1	10.8^b^	6
	7 (non-V600E)		13.7	71	8.9	15.2	
Platinum-based doublet chemotherapy^c^	23 (V600E and non-V600E)	Treatment-naive	NA	NA	6.4	18.4	104
Immunotherapy^d^	12 (V600E)	Treatment-naive and previously treated	5.5	25	3.7	NE	76
	10 (non-V600E)			33^e^	4.1	NE	
Immunotherapy^f^	17 (V600E)	Treatment-naive and previously treated	16.1	24	1.8	8.2	77
	18 (non-V600E)				4.1	17.2	
Immunotherapy^g^	26 (V600)	Treatment-naive and previously treated	9.2	26	5.3	22.5	23
	18 (non-V600)			35	4.9	12.0	
Immunotherapy^h^	8 (V600E)	Treatment-naive	NA	38	10.5	NA	105
	7 (non-V600E)			43	10.8	NA	
Immunotherapy as monotherapy or in combination^i^	43 (V600E)	Treatment-naive and previously treated	16.2	51.7	10.0	18.5	83
	16 (non-V600E)			31.1	8.0	16.0	
Immunotherapy-combined chemotherapy^j^	9 (V600E)	Treatment-naive	NA	56	18.5	NA	82
	7 (V600E)	Previously treated	NA	29	1.9	NA	

*NA* not available, *NE* not estimable, *ORR* objective response rate, *OS* overall survival, *PD-1* programmed cell death protein 1, *PD-L1* programmed death ligand 1, *PFS* progression-free survival.

^a^All patients received platinum-based doublet combination chemotherapy.

^b^Median OS was calculated for *n* = 12 in each group.

^c^All patients received platinum-based doublet chemotherapy (most commonly, carboplatin-pemetrexed with or without bevacizumab, but several patients received cisplatin instead of carboplatin and docetaxel or etoposide instead of pemetrexed).

^d^Includes nivolumab (*n* = 11), pembrolizumab (*n* = 10), and atezolizumab (*n* = 1).

^e^ORR for non-V600E was calculated out of 9 patients.

^f^Most patients (94%) in the full study received anti-PD-1 antibodies (nivolumab [*n* = 466], pembrolizumab [*n* = 48], other [*n* = 6]) or anti-PD-L1 antibodies (atezolizumab [*n* = 19], durvalumab [*n* = 11], other [*n* = 1]). The treatment breakdown for specifically the BRAF-mutant cohort was not reported.

^g^For the V600 cohort, this includes nivolumab (*n* = 18), pembrolizumab (*n* = 6), and other (*n* = 2). For the non-V600 cohort, this includes nivolumab (*n* = 16) and pembrolizumab (*n* = 2).

^h^Primarily pembrolizumab either as monotherapy or in conjunction with chemoimmunotherapy for three of the non-V600E patients.

^i^Specific immunotherapy treatments were not provided. Of the patients with BRAF-mutant NSCLC (*n* = 59), 30.5% received immunotherapy monotherapy, 62.7% received immunotherapy plus chemotherapy, and 6.8% received immunotherapy plus anti-angiogenesis.

^j^Specific therapies were not specified.

### Chemotherapy

Prior to the development of targeted therapy for BRAF^V600E^-mutant metastatic NSCLC, platinum-based chemotherapy was the standard of care^68,78^. However, retrospective studies reported that patients with BRAF^V600E^-mutant NSCLC had poorer outcomes with platinum-based chemotherapy than those with BRAF^WT^ NSCLC (Table 2)^6,32^. While those retrospective studies also reported a shorter PFS in patients with V600E mutations (4.1–5.2 months) versus non-V600E mutations (6.4–8.9 months), another retrospective study reported that carboplatin-pemetrexed in patients with treatment-naive BRAF-mutant metastatic NSCLC resulted in longer PFS in patients with class I mutations (6.2 months) versus class II or III mutations (3.3 months and 4.9 months, respectively)^33^. Additionally, in a multi-institutional prospective lung cancer screening project, median PFS with platinum-containing chemotherapy was longer in patients with class I mutations (11.5 months) than in those with class III mutations (5.3 months)^31^. Several trials demonstrated that BRAF monotherapy or BRAF plus MEK inhibitor therapy was effective in patients who had progressed on chemotherapy^10,15,18,66^. Chemotherapy remains a second-line recommendation for patients with a BRAF^V600^ mutation^21,22^.

### Immunochemotherapy

The combination of immunotherapy and chemotherapy is approved for first-line treatment of patients with metastatic NSCLC but not specifically for patients with BRAF^V600E^ mutations^80^. In a phase 3 trial of treatment-naive patients with metastatic non-squamous NSCLC, the combination of pembrolizumab, pemetrexed, and a platinum resulted in significantly longer median PFS compared with chemotherapy alone (8.8 versus 4.9 months; HR = 0.52; *P* < 0.001)^81^. In a small retrospective study in China, immunotherapy plus chemotherapy (*n* = 9) in treatment-naive patients with BRAF^V600E^-mutant advanced NSCLC resulted in a significantly longer median PFS compared with chemotherapy or targeted therapy (*n* = 20; 18.5 versus 4.1 months; *P* = 0.0098)^82^. This efficacy benefit with immunochemotherapy was not observed in later lines. Another retrospective study showed similar efficacy with immunotherapy-based treatments in patients with advanced NSCLC with or without *BRAF* mutations; median PFS was 8.4 months in both patient populations^83^. In the BRAF cohort, median PFS was similar for V600E and non-600E mutations (10.0 versus 8.0 months). Median PFS was longer in the first line than in subsequent treatment lines in patients with WT (12.8 versus 5.6 months) and BRAF-mutant (11.2 versus 4.0 months) NSCLC. These studies suggest that immunotherapy-based treatments are an option for patients with BRAF^V600E^-mutant advanced NSCLC^82,83^.

## Emerging treatments and approaches

### Immunotherapy plus targeted therapy combinations

The combination of BRAF-targeted therapy plus immunotherapy may produce a synergistic anti-tumor effect; tolerability of the combined approach will be a key consideration^14^. Studies have investigated various combinations of anti-PD-1/PD-L1 with BRAF and/or MEK inhibitors and reported positive outcomes in solid tumors, including BRAF^V600E/K^-mutant melanoma, CRC, and BRAF^V600E^-mutant NSCLC^84–87^. In a phase 1/1b, global, multicenter, open-label study, cobimetinib and atezolizumab (anti-PD-L1) were evaluated in immunotherapy-naive patients with advanced solid tumors (*n* = 150); patients had received a median of 5.0 prior systemic therapies, and 15% had *BRAF* mutations^84^. In patients with metastatic CRC (*n* = 84), ORR was 8% (95% CI, 3–16%), median PFS was 1.9 months (95% CI, 1.8–2.3), and median OS was 9.8 months (95% CI, 6.2–14.1). In patients with melanoma (*n* = 22), ORR was 41% (95% CI, 21–64%), median PFS was 13.3 months (95% CI, 2.8–NE), and median OS was NE (95% CI, 18.7–NE). In patients with NSCLC (*n* = 28), ORR was 18% (95% CI, 6–37%), median PFS was 5.8 months (95% CI, 2.7–9.2), and median OS was 13.2 months (95% CI, 9.2–NE). In the safety analysis (*n* = 150), the most common TRAEs were diarrhea (67%), rash (48%), and fatigue (40%); 44% of patients reported grade 3–4 TRAEs. Another trial (NCT03991819) is evaluating the combination of binimetinib and pembrolizumab in patients with *EGFR* WT, *ALK*-rearrangement–negative advanced or metastatic NSCLC with PD-L1 tumor proportion score (TPS) ≥ 50%; initial results reported that 33% of nine evaluable patients had a partial response, including one patient with BRAF^V600E^-mutant metastatic NSCLC^85,86^.

Trials have investigated the efficacy and safety of triple combination therapies for BRAF-mutant melanomas^87–89^. A phase 2, randomized trial enrolled patients with treatment-naive advanced melanoma with a V600E or V600K mutation to receive dabrafenib plus trametinib with or without pembrolizumab (triplet, *n* = 60; doublet, *n* = 60)^88^. When compared with the doublet therapy, triplet therapy resulted in longer median PFS (10.7 versus 16.9 months) and higher incidence of grade ≥3 TRAEs (25.0% versus 58.3%). Grade ≥3 AEs occurred in 70% of patients in the triplet arm and 45% of patients in the doublet arm. Immune-mediated AEs occurred in 15% and 52% of patients in the doublet and triplet treatment arms, respectively; pneumonitis (17%) and hypothyroidism (8%) were the most common immune-mediated AEs reported with triplet therapy. An open-label, phase 1/2 trial combined encorafenib plus binimetinib with pembrolizumab for patients with BRAF^V600^-mutant advanced melanoma (*n* = 15); ORR was 64% (95% CI, 35–87%), and 12-month PFS was 41% (95% CI, 13–68%)^87^. TRAEs were reported by 87% of patients; grade ≥3 TRAEs were reported in 53% of patients, with increases in aspartate aminotransferases, gamma glutamyl transferase, and creatine phosphokinase being the most common. A meta-analysis of triplet therapies compared with doublet therapy or monotherapy for melanoma revealed that triplet therapy significantly improved PFS and OS but was associated with increased frequency of immune-related AEs, including hypothyroidism, arthralgia, myalgia, alanine aminotransferase elevations, aspartate aminotransferase elevations, asthenia, and pyrexia^90^. Triplet therapy did not increase the overall incidence of AEs or grade ≥3 AEs. The increased incidence of AEs should be considered when determining the optimal combination of immunotherapy and targeted therapy^88,90^.

### Next-generation BRAF inhibitors

Next-generation BRAF inhibitors target dimerization since it is an essential component of activation for WT and many mutant BRAF kinases, plays a role in resistance mechanisms to BRAF inhibitors, and is associated with AEs^91^. These drugs were developed following two main strategies aimed at inhibiting mutant RAF while preventing paradoxical activation and common acquired resistance mechanisms^92^. Type II pan-RAF inhibitors bind the active conformation of RAF monomers and dimers^92,93^. Despite the name of pan-RAF, at least three of these agents (naporafenib [LXH254], tovorafenib [TAK-580], belvarafenib) demonstrated poor inhibition of ARAF and potent inhibition of WT and mutant versions of CRAF and BRAF^93^. Paradox breakers (e.g., PLX8394, PLX7904) are more specific BRAF inhibitors that alter the dimer interface and subsequently prevent BRAF-homodimer and BRAF:CRAF heterodimer formations^92,94^. Data from preclinical and clinical studies suggest that *BRAF* non-V600 mutations could be targeted with these new inhibitors, and several are being investigated in ongoing trials to better define their efficacy and safety (Table 3)^34,95,96^.

**Table 3:** Ongoing trials and initial results of emerging BRAF inhibitors

Treatment	Trial	Phase	Patients	Actual/estimated enrollment	Primary outcomes	Ref
KIN-2787	Preclinical	Biochemical	BRAF-mutant human cancer models	–	Nanomolar to picomolar potency against RAF1, BRAF, and ARAF (IC_50_: 0.06–3.46 nM)	106
		Cell-based assays	BRAF-mutant human cancer models	–	Class II and III BRAF-mutant cell lines were the most responsive; 19- and 7-fold more sensitive than cells with WT BRAF, respectively	
KIN-2787 ± binimetinib	NCT04913285	1/1b	BRAF- and/or NRAS-mutant solid tumors; BRAF and MEK inhibitor-naive for phase 1b	262	Phase 1a: MTD and DLTs Phase 1b: ORR, DCR, DOR, and duration of SD	107
Naporafenib (LXH254) + LTT462 or trametinib or ribociclib	NCT02974725	1b	KRAS- or BRAF-mutant advanced or metastatic NSCLC; NRAS-mutant cutaneous melanoma	242 (actual)	Number of patients with AEs, DLTs, tolerability measured by number and reasons for interruptions/reductions, dose intensity of study drug	108
	Initial results	Escalation	KRAS- or BRAF-mutant advanced or metastatic NSCLC; NRAS-mutant melanoma	36	6 patients reported grade ≥3 DLTs, including dermatitis acneiform (*n* = 2), maculopapular rash (*n* = 2), increased lipase (*n* = 1), and Stevens-Johnson syndrome (*n* = 1)	95
		Expansion	NRAS-mutant melanoma	Cohort 1 (*n* = 15): received naporafenib 200 mg twice daily + trametinib 1 mg once daily Cohort 2 (*n* = 15): received naporafenib 400 mg twice daily + trametinib 0.5 mg once daily	Safety (*n* = 30) 100% reported a TRAE; most common was rash (80%) Efficacy: Cohort 1: ORR was 46.7% (95% CI, 21.3–73.4), median DOR was 3.75 months (95% CI, 1.97–NE), median PFS was 5.52 months Cohort 2: ORR was 13.3% (95% CI, 1.7–40.5), median DOR was 3.75 months (95% CI, 2.04-NE), and median PFS was 4.21 months	95
Tovorafenib (TAK-580)	NCT01425008^a^	1	Relapsed or refractory advanced solid tumors	Total: 149 Cohort 1: targeted therapy-naive BRAF-mutant receiving Q2D in expansion phase (*n* = 16) Cohort 2: targeted therapy previously treated BRAF-mutant receiving Q2D in expansion phase (*n* = 6)	Safety (*n* = 149): 32.9% grade ≥3 TRAEs Most common TRAEs: fatigue (37.6%) and anemia (23.5%) SCC was reported as TEAE by 1 patient Efficacy: For BRAF-mutant tumors, ORR was 50% and 17% for cohorts 1 and 2, respectively	96,109
Tovorafenib (TAK-580) ± pimasertib	NCT04985604	1b/2	Recurrent, progressive, or refractory melanoma or other solid tumors (including NSCLC) with mutations in MAPK pathway	168	Phase 1b: Determine safety (AEs), MTD, and RP2D of tovorafenib + other therapies Phase 2: Efficacy of tovorafenib ± other therapies (ORR)	110
HLX208 (BRAF inhibitor)	NCT05065398	2	BRAF and MEK inhibitor-naive BRAF^V600^-mutant advanced NSCLC	20	ORR	111
HLX208 + HLX10 (anti-PD-1 antibody)	NCT05641493	1b/2	BRAF^V600E^-mutant advanced solid tumors (phase 1b) or NSCLC (phase 2) with positive PD-L1 expression (TPS or TC ≥ 1%); BRAF and MEK inhibitor-naive (phase 2)	49	Phase 1b: MTD and DLT Phase 2: ORR	112

*AE* adverse event, *DCR* disease control rate, *DLT* dose-limiting toxicity, *DOR* duration of response, *IC*_*50*_ half maximal inhibitory concentration, *MTD* maximum tolerated dose, *NE* not estimable, *NSCLC* non-small cell lung cancer, *ORR* objective response rate, *PFS* progression-free survival, *Q2D* once every other day, *RP2D* recommended phase 2 dose, *SCC* squamous cell carcinoma, *SD* stable disease, *TC* tumor cell, *TEAE* treatment-emergent AE, *TPS* tumor proportion score, *TRAE* treatment-related AE, *WT* wild type.

^a^Phase 1 was completed in 2018.

## Unmet needs

### Brain metastases

Brain metastases (BMs), a common (26% at diagnosis) challenge for patients with metastatic NSCLC, are associated with poor prognosis and quality of life^97,98^. One study reported that the incidence of baseline BMs in patients with BRAF-mutant metastatic NSCLC was significantly lower for class I mutations (9%) than for class II (29%; *P* = 0.011) or class III (31%; *P* = 0.007) mutations^33^. However, this subset of patients has been predominantly excluded from trials of targeted therapies^15,16,18^. In a trial evaluating vemurafenib in patients with BRAF-mutant advanced NSCLC, median PFS was 1.9 months (95% CI, 1.5–3.9) and 5.4 months (95% CI, 3.8–7.2) in patients with (*n* = 26) and without (*n* = 89) baseline BMs^11^. In patients enrolled in PHAROS with BRAF^V600E^-mutant metastatic NSCLC asymptomatic BMs, ORR was 100% (95% CI, 39.8–100.0%) for treatment-naive patients (*n* = 4) and 0% for previously treated patients (*n* = 4)^18^. Better understanding of the epidemiology, risk, impact, and treatment of BMs in patients with BRAF-mutant metastatic NSCLC remains an unmet need.

More robust efficacy analysis of patients with BMs has been conducted for BRAF-mutant metastatic melanoma treated with BRAF/MEK inhibitors. In a phase 3 study of patients with BRAF^V600^-mutant unresectable or metastatic melanoma with BMs (*n* = 275), systemic outcomes with dabrafenib plus trametinib were ORR of 41.5% and median PFS of 5.68 months (95% CI, 5.29–6.87)^99^. Treatment-naive patients had a significantly longer median PFS than previously treated patients (7.23 versus 4.96 months; *P* = 0.0061). An open-label, multi-cohort, phase 2 study evaluated the efficacy of dabrafenib plus trametinib in patients with BRAF^V600^-mutant melanoma with BMs (*n* = 125), including the largest cohort (*n* = 76) with asymptomatic BRAF^V600E^-mutant BMs and no prior brain-directed therapy^100^. For the largest cohort, intracranial response rate (IC ORR) was 58% (95% CI, 46–69%), median PFS was 5.6 months (95% CI, 5.3–7.4), and median OS was 10.8 months (95% CI, 8.7–19.6). The safety profile was consistent with those in previous dabrafenib plus trametinib studies. In a multicenter, retrospective case series investigation, encorafenib plus binimetinib combination treatment of 24 patients with BRAF-mutant metastatic melanoma BMs resulted in IC ORR of 33% and ORR of 21%^101^. The safety profile was consistent with what was reported in patients with melanoma without BMs.

## Conclusion

BRAF^V600E^ is an actionable mutation for metastatic NSCLC with safe and effective treatment options^8,16,18^. BRAF and MEK inhibitor combination therapies have demonstrated rapid and durable responses in the majority of patients^15,16,18^. The safety profiles of these combinations are well described, and appropriate therapy management principles should be employed for responding patients. Disease progression is inevitable, as only ~50% of patients with metastatic NSCLC receive a second-line treatment^102,103^. Therefore, it is prudent to use the most efficacious agents in the first-line setting. Further studies are necessary to determine optimal sequencing methods, understand resistance mechanisms, determine efficacy of treatments for BMs, and develop targeted therapies for non-V600E mutations.

### Reporting summary

Further information on research design is available in the Nature Research Reporting Summary linked to this article.

### Supplementary information

REPORTING SUMMARY

## Data Availability

No datasets were generated or analyzed for this article. Data referenced in this review can be accessed by following resources numbered in the References section.
