# Author Correction: BRAF^V600E^-mutant metastatic NSCLC: disease overview and treatment landscape

**DOI:** 10.1038/s41698-024-00633-7

**Published:** 2024-07-15

**Authors:** David Planchard, Rachel E. Sanborn, Marcelo V. Negrao, Aria Vaishnavi, Egbert F. Smit

**Affiliations:** 1 Thoracic Cancer Group, Department of Medical Oncology, Gustave Roussy, Villejuif, France; 2 Earle A. Chiles Research Institute, Providence Cancer Institute, Portland, OR USA; 3 Department of Thoracic/Head and Neck Medical Oncology, Division of Cancer Medicine, University of Texas MD Anderson Cancer Center, Houston, TX USA; 4 Department of Cancer Biology, University of Texas MD Anderson Cancer Center, Houston, TX USA; 5 Department of Pulmonary Disease, Leiden University Medical Centre, Leiden, Netherlands

**Keywords:** Non-small-cell lung cancer, Targeted therapies

Correction to: *npj Precision Oncology* 10.1038/s41698-024-00552-7, published online 16 April 2024

In the Acknowledgements section, the funding source was omitted. The original article has been corrected.

